# One million cyclable blue/colourless electrochromic device using K_2_Zn_3_[Fe(CN)_6_]_2_ nanoparticles synthesized with a micromixer†

**DOI:** 10.1039/c9ra09496b

**Published:** 2019-12-12

**Authors:** Akira Takahashi, Keiko Noda, Hiroshi Watanabe, Tohru Kawamoto

**Affiliations:** Nanomaterials Research Institute, National Institute of Advanced Industrial Science and Technology (AIST) 1-1-1 Higashi Tsukuba Ibaraki 305-8565 Japan tohru.kawamoto@aist.go.jp akira-takahashi@aist.go.jp

## Abstract

Well-defined K_2_Zn_3_[Fe(CN)_6_]_2_-nanoparticles (NPs) synthesized with a micromixer showed robust redox reaction cyclability. Crystal structure analysis revealed that the robustness results from the maintenance of the original rhombohedral crystal structure in the oxidation state. The blue/colourless electrochromic device with the K_2_Zn_3_[Fe(CN)_6_]_2_-NPs and Prussian blue NPs showed recyclability over 1 million redox reactions.

## Abstract 2

Well-defined K_2_Zn_3_[Fe(CN)_6_]_2_-nanoparticles (NPs) synthesized with a micromixer showed robust redox reaction cyclability.

## Graphical abstract

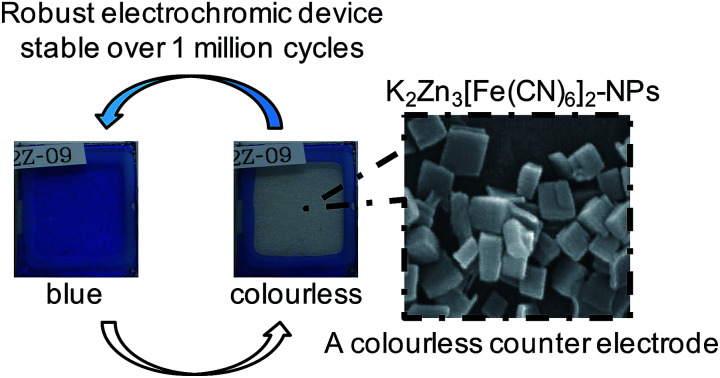

Electrochromic devices (ECDs), otherwise known as colour-switching devices, produced by electrochemical redox reactions, are attracting attention owing to their several applications in various systems such as in electric paper display devices^1–3^ and smart window systems for the external control of light and heat flow.^4–7^ Several types of materials have been reported for use in ECDs,^8^*e.g.* Prussian blue analogues,^9–12^ tungsten oxide,^13–15^ and organic materials, including π-conjugated organic polymers.^16^

When these materials are used as working electrodes in ECDs, the application of electrochemically active material for a counter electrode is also essential. Particularly, if the ECDs are used for smart windows or transparent displays, the counter electrode material must satisfy either of the following requirements: it must be transparent for visible light in both the redox states or show complementary reaction with the working electrode. For example, potassium zinc hexacyanoferrate (K_2_Zn_3_[Fe(CN)_6_]_2_, KZnHCF), a Prussian blue analogue, was found to have high transmittance in the visible region at both the oxidation (Zn^II^_3_[Fe^III^(CN)_6_]_2_) and reduction states (K_2_Zn^II^_3_[Fe^II^(CN)_6_]_2_).^17^ In addition, the KZnHCF nanoparticles (NPs) show a relatively high cyclic stability in ECD application.^18^ In this case, KZnHCFs were prepared using classification to pick up small NPs, implying that the yield was rather low. In this study, to increase the yield to prepare the well-defined KZnHCF NPs, we developed a new synthesis method of KZnHCFs using a micromixer. Using the KZnHCF-NPs synthesized with the micromixer, high cyclic stability was achieved with over 1 million runs using the KZnHCF-NPs as the counter electrode in ECDs in combination with the Prussian blue NPs as the working electrode.

KZnHCF-NPs were synthesized with the micromixer and by a batch method by mixing the same volume of 4 mmol L^−1^ of K_4_[Fe(CN)_6_] aqueous solution and 6 mmol L^−1^ of ZnCl_2_ aqueous solution and adjusting the pH to 5 using HCl and NaOH (hereinafter referred to as the standard concentration). The reaction is expressed by the following equation:3ZnCl_2_ + 2K_4_[Fe(CN)_6_] → K_2_Zn_3_[Fe(CN)_6_]_2_ + 6KCl

The temperatures of the solutions before and after utilising the micromixer were adjusted from 8 to 10 °C using a 10 m precooling tube consisting of an internal diameter (i.d.) of 0.5 mm stainless steel (SUS304) and a 100 m reaction tube consisting of an i.d. of 3 mm silicon tube, respectively (hereinafter referred to as the standard condition), as shown in Fig. 1(a). To evaluate the effectiveness of the micromixer, the dependence of the particle size on the flow rate was investigated. Subsequently, KZnHCF was synthesized by mixing standard concentrations of ZnCl_2_ and K_4_[Fe(CN)_6_] aqueous solutions with the total flow rates set to 10, 40, 100, and 140 mL min^−1^, separately. The linear velocities in the micromixer were 9.4, 38, 94, and 132 m s^−1^ at 10, 40, 100, and 140 mL min^−1^, respectively. The batch sample was synthesized by mixing 50 mL of each reagent, whose solutions were the same as the standard solutions in a beaker stirred and cooled in ice water. The dependence of the reagent concentration on the NP size was evaluated in the standard concentration and at 132 m s^−1^ with different concentrations, where the concentrations of ZnCl_2_ were 0.60, 2.0, 4.0, 6.0, 60, and 600 mmol L^−1^, and the concentrations of K_4_[Fe(CN)_6_] were 0.40, 1.32, 2.67, 4.0, 40, and 400 mmol L^−1^.

**Fig. 1:**
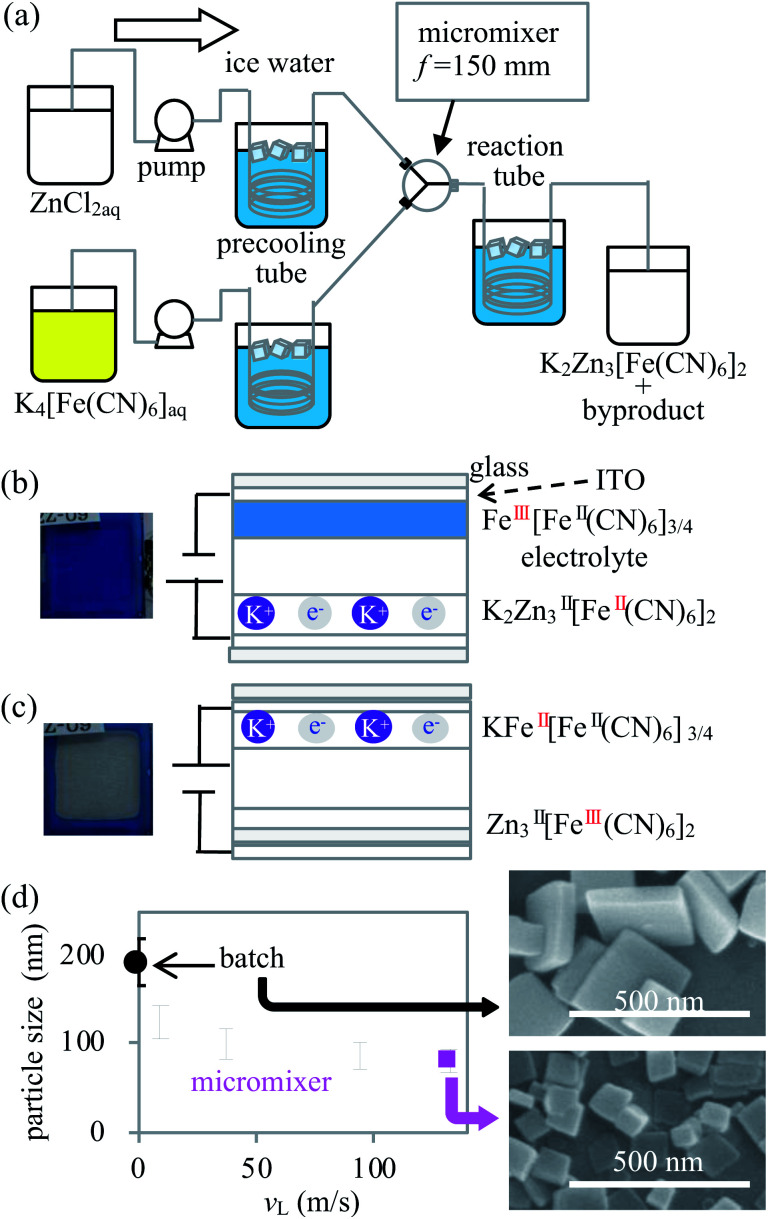
(a) Apparatus for the KZnHCF synthesis using a micromixer; (b) cross-sectional image of the ECD in blue colour, (c) that of the colourless ECD, and (d) the relationship between the particle size of KZnHCFs and the flow speed in the micromixer. Batch KZnHCF is indicated at 0 m s^−1^.

The particle morphology was evaluated using a scanning electron microscope (SEM, S-4800, Hitachi High-Technologies Corp.). The particle size was evaluated based on the longest diagonal distance in the SEM figure. The average distribution of the particle size was evaluated with considering more than 100 particles per sample.

The crystal structure of KZnHCFs was evaluated by powder X-ray diffraction (PXRD, D2 phaser, Bruker Corp.). A KZnHCF thin film was prepared on a stainless steel (SUS-304) substrate using the spin-coating method. The crystal structure of the sample was evaluated by Rietveld refinement using DIFFRAC.TOPAS 5 (Bruker Corp.). The experimental details of the film preparation are provided in ESI.† The crystal structure of the oxidised state of KZnHCF was analysed after the electrochemical oxidation, as shown in ESI.†

Three terminal electrochemical evaluations of the KCuHCF electrode were made as follows: the KZnHCF thin films on an indium tin oxide (ITO) glass substrate were also fabricated by spin coating under the conditions of 1000 rpm for 10 s on the 2.5 cm × 2.5 cm ITO glass substrate. The area of the working electrode was set to 1.0 cm^2^. A propylene carbonate (PC) solution comprising 0.1 mol L^−1^(PC) of potassium bis(trifluoromethanesulfonyl) imide, saturated calomel electrode (SCE), and platinum wire, were used as the electrolyte, the reference electrode, and the counter electrode, respectively. The electrochemical behaviour was analysed using an electrochemical analyser (ALS6115D BAS inc.). The cyclic voltammogram (CV) was obtained at a scanning speed of 5 mV s^−1^ and a potential range of 0.4–1.4 V (*vs.* SCE). The ultraviolet-visible (UV-vis) spectra of the samples were obtained using a UV-vis spectrometer (USB-4000, ocean optics corp.) after the spin-coating process on the ITO glass substrate.

The ECDs were fabricated by sandwiching the electrolyte between the Prussian blue film on the ITO glass and the KZnHCF film on the ITO glass. The detailed method for the fabrication and preparation of the ECDs are described in ESI.† For the electrolyte, the propylene carbonate solution containing 0.1 mol L^−1^ of potassium bis(trifluoromethane sulfonyl)imide and 360 g L^−1^ of polymethyl methacrylate was used. The water-dispersible ink of Prussian blue NPs (Fe^III^[Fe^II^(CN)_6_]_3/4_) was purchased from Kanto-chemical co., inc. The electrochemical reactions at each electrode are detailed below.K_2_Zn^II^_3_[Fe^II^(CN)_6_]_2_ (colourless) → Zn^II^_3_[Fe^III^(CN)_6_]_2_ (colourless)+ 2K^+^ + 2e^−^Fe^III^[Fe^II^(CN)_6_]_3/4_ (Blue) + K^+^ + e^−^ → KFe^II^[Fe^II^(CN)_6_]_3/4_ (colourless)where KZnHCF acts as the colourless counter electrode (Fig. 1(b) and (c)).

The cyclic stability test of the ECDs was performed for each ECD consisting of KZnHCFs under the following condition. The voltage was set at −0.3 V for 4 s and at −1.2 V for 2 s cyclically with the use of an electro power supply (GS200, Yokogawa Denki) for 1 million cycles. To evaluate the performance change of the ECDs in the cyclic stability test after 110 000 600 000, and 1 000 000 cycles, the performances of the ECD samples were evaluated by chronocoulometry processes and UV-vis spectroscopy. In chronocoulometry processes 1 and 2, the voltage was set from −0.3 V to −1.2 V (CC1) and from −1.2 V to −0.3 V (CC2), respectively, and the potential was maintained for 30 s. Five samples of each KZnHCF were taken and their averages were evaluated.

First, we investigated the KZnHCF NP samples synthesized with different linear velocities, *v*_L_, by SEM. The averages and standard deviations of the particle size were 124 ± 38, 99 ± 34, 88 ± 29, and 80 ± 24 nm for *v*_L_ = 9.4, 37, 94, and 132 m s^−1^, respectively. In the case of batch synthesis, the particle size was 192 ± 50 nm. The averages and dispersions of the particle size decreased as *v*_L_ increased, as shown in Fig. 1(d). The particle size distributions in each linear velocity are shown in ESI.† In general, the micromixer synthesis with a relatively high *v*_L_ provides relatively small and homogeneous NPs due to intense and rapid mixing in the micromixer.^19^ The mixed flow in the micromixer, except for the case of *v*_L_ = 9.4 m s^−1^, was considered to be turbulent because the Reynolds number was greater than 3000. Particularly, 86% of the particles synthesized at the highest linear velocity (*v*_L_ = 132 m s^−1^) are smaller than 100 nm.

We discovered that the reagent concentrations have a strong impact on the morphology and particle size of KZnHCF. The concentration of ZnCl_2_, *c*_Zn_, was set to be 1.5 times higher than that of K_4_[Fe(CN)_6_], *c*_Fe_. The dependence of the particle size on *c*_Zn_ is shown in Fig. 2. The particle size had minimal values, 2.0 < *c*_Zn_ < 6.0 mmol L^−1^. In the case of *c*_Zn_ < 0.6 mmol L^−1^, some KZnHCF NPs had various polyhedral shapes. Such shapes may not be suitable for ECDs made by the coating method, because the polyhedron corn prevents particle interactions, implying weak electron migration. Conversely, in the case of *c*_Zn_ > 6.0 mmol L^−1^, very large particles were obtained, which are also unsuitable for ECDs due to the slow ion migration in the particles. As a result, we conclude that 2.0 < *c*_Zn_ < 6.0 mmol L^−1^ would be suitable for the ECD. In particular, for *c*_Zn_ = 6.0 mmol L^−1^, the synthesis yield of KZnHCF was beyond 90%. Conversely, there was no dependence on the tube length in the reactor.

**Fig. 2:**
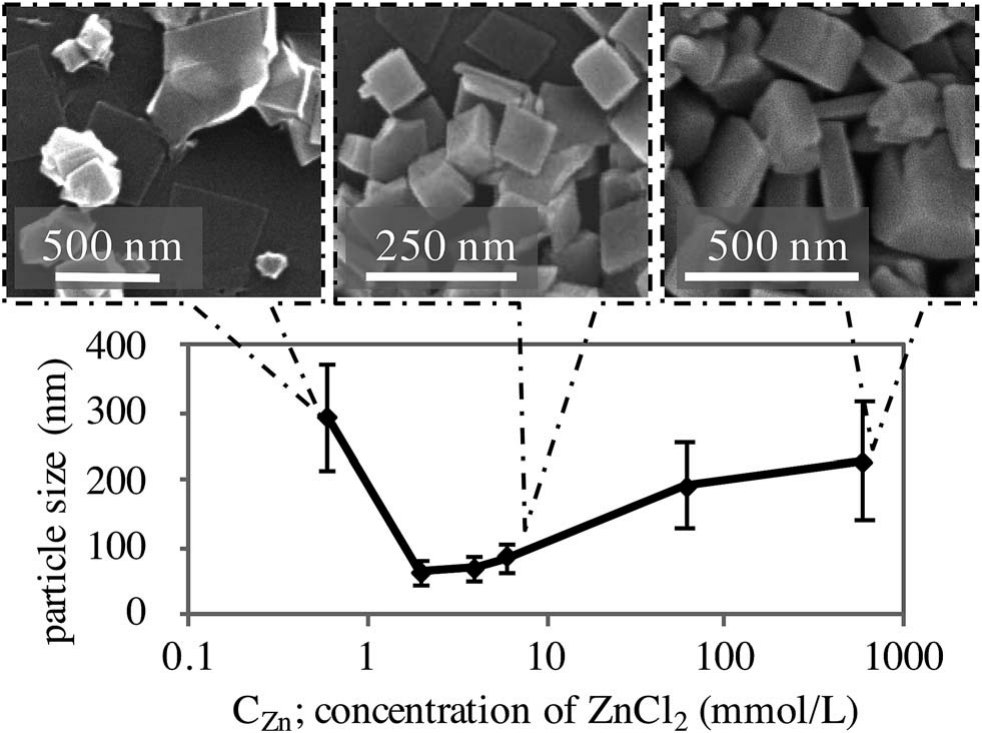
Influence of concentration on the particle size and SEM figure of KZnHCFs synthesized with each *C*_Zn_.

We also evaluated the influence of temperature on the particle size. The relatively high temperature provided relatively large particle sizes of KZnHCF (See Fig. S1†). The cooling condition with ice water is better for synthesizing the KZnHCF NPs.

For the detailed studies, the KZnHCFs synthesized using the two methods were compared. The batch method (KZnHCF-B) and the micromixer method (KZnHCF-M) with RF = 132 m s^−1^ and *c*_Zn_ = 6 mmol L^−1^. The as-synthesized KZnHCF-B has low dispersibility in water and could not be well coated on the ITO glass substrate with aggregations. To make KZnHCF-B dispersible, we conducted surface treatment with a K_4_[Fe(CN)_6_] solution in accordance with previous studies.^20^ Conversely, KZnHCF-M was sufficiently dispersible for the fabrication of the thin film by spin-coating. The obtained KZnHCFs were modified with these K_4_[Fe(CN)_6_] surfaces to increase their dispensability^21,22^ in aqueous solution as shown in ESI.† The surface-treated KZnHCFs were represented as KZnHCF-BS and KZnHCF-MS.

The chemical compositions, including the hydration number and crystal structures of these KZnHCFs, were evaluated. The methods of which are shown in ESI.† The chemical compositions were as follows: KZnHCF-M: K_1.8_Zn_3.0_[Fe(CN)_6_]_1.9_·6.9H_2_O, KZnHCF-MS: K_1.9_Zn_3.0_[Fe(CN)_6_]_1.9_·7.2H_2_O, and KZnHCF-BS: K_2.0_Zn_3.0_[Fe(CN)_6_]_2.0_·6.8H_2_O, as shown in Table S1.† The number of the surface sites of KZnHCFs is much lower than that of [Fe(CN)_6_] in the crystal structure; the surface treatment caused a slight difference in the chemical composition. The PXRD patterns are consistent with the previously reported structure of Na_2_Zn_3_[Fe(CN)_6_]_2_, and the space group is *R*3̄*c*,^23^ as shown in Fig. S4(b).†

The electrochemical properties of the thin films of KZnHCF-BS and KZnHCF-MS on the ITO glass substrates were investigated with the three-terminal electrode method. Their CV curves showed nearly similar profiles with two pairs of redox peaks, as shown in Fig. 3(a). The main peak pairs correspond to the redox reaction of [Fe^II/III^(CN)_6_] constructing a porous network in KZnHCF. The second peak could be caused by the redox reaction of the remaining K_4/3_[Fe^II/III^(CN)_6_] surface. The chronocoulometry curves of KZnHCF-BS and KZnHCF-MS were obtained as shown in Fig. 3(b). The injected charge density of the KZnHCF-BS thin film was 5.3 mC cm^−2^ and that of KZnHCF-MS was 6.5 mC cm^−2^.

**Fig. 3:**
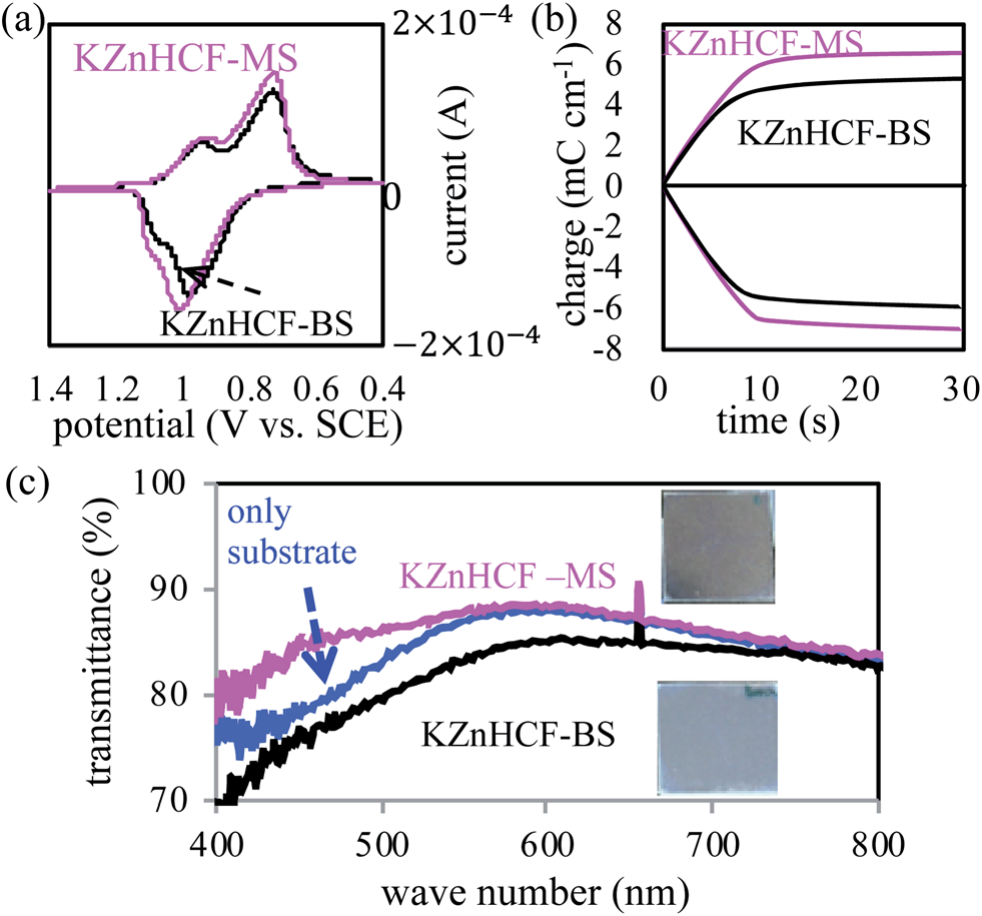
(a) Cyclic voltammetry of the KZnHCF-BS and KZnHCF-MS electrodes, (b) chronocoulometry curve of the KZnHCF-BS and KZnHCF-MS electrodes, and (c) UV-vis spectra of KZnHCF-BS on substrate, KZnHCF-MS, and only ITO glass.

The UV-vis spectra of the KZnHCF-BS and KZnHCF-MS electrodes were also measured. The transmittance of the KZnHCF-MS electrode on the ITO glass is higher than that of the KZnHCF-BS electrode on the ITO glass as shown in Fig. 3(c). The low transmittance of KZnHCF-BS could be caused by the scattering of the relatively large particles. The high transmittance of the KZnHCF-MS electrode indicates less surface roughness even in comparison with the bare ITO substrate.

The crystal structures of the electrochemically oxidised states of KZnHCF-MS were evaluated by PXRD and Rietveld refinement. The XRD pattern of the oxidised and reduced states shown in Fig. S4(a)† indicates the same space groups, *R*3̄*c*, as the initial (reduced) state with the minor changes in the lattice constant, *i.e. a* = 12.54 Å and *c* = 32.08 Å for the initial state, and *a* = 12.45 Å and *c* = 33.35 Å for the oxidised state. This result indicates that we succeeded in making the new phase electrochemically different from the structure synthesized by precipitation method, *Fm*3̄*m*.^24^

The blue/colourless ECDs consisting of Prussian blue and KZnHCF NPs (details in ESI†) are consistent with those of previous research.^20^ The ECD has a blue colour with a reduced state of KZnHCF(K_2_Zn^II^_3_[Fe^II^(CN)_6_]_2_) and an oxidised state of Prussian blue (Fe^III^_4_[Fe^II^(CN)_6_]_3_) which has large absorbance at the peak of 700 nm due to the metal to metal charge transfer, as shown in Fig. 4(c). Conversely, the ECD is transparent with an oxidised state of KZnHCF(Zn^II^_3_[Fe^III^(CN)_6_]_2_) and a reduced state of Prussian blue (KFe^II^_4_[Fe^II^(CN)_6_]_3_), which is known as Prussian white owing to its low absorbance in the visible region.

**Fig. 4:**
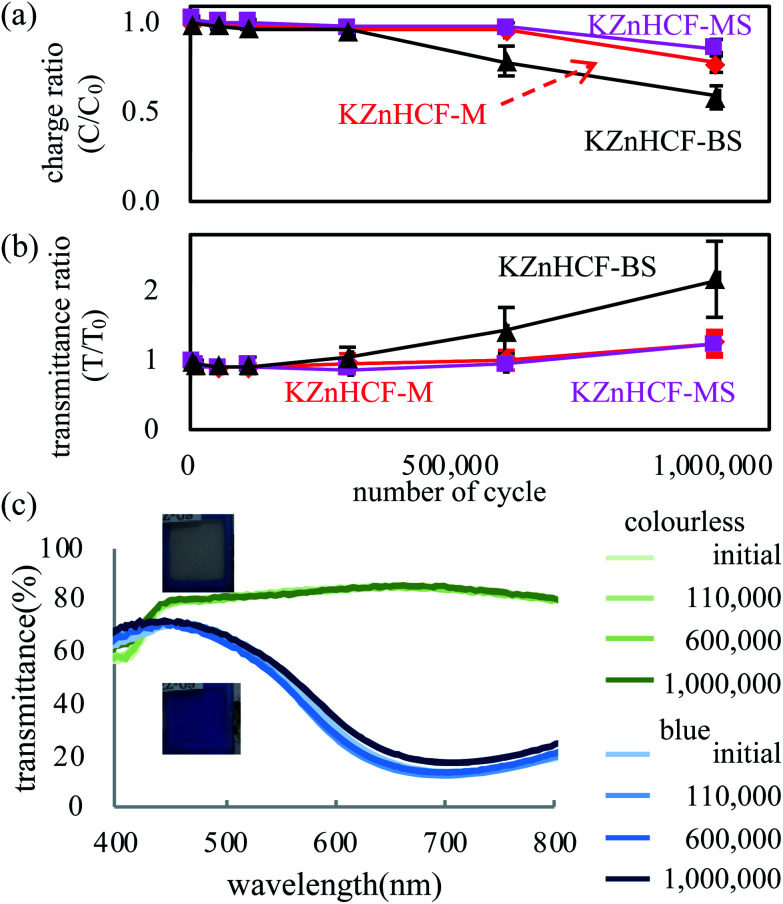
(a) Effective charge and transmittance ratios to the initial value in the cyclic test of the PB-KZnHCF ECD, and (b) UV-vis spectra in the cyclic test.

Concerning the colour-switching speed, we evaluated the reaction time for colouration, *t*_C_, and for bleaching, *t*_B_. These indicators are defined as the time required for 80% of transmittance change from the initial state to the completely switched states of CC1 and CC2.^18^ For colouration, we evaluated *t*_C_ = 1.1, 1.0, and 1.3 s for the ECDs using KZnHCF-MS, KZnHCF-M, and KZnHCF-BS, respectively, while *t*_B_ = 1.3, 3.0, and 3.8 s for each ECD as shown in ESI.† The result shows that both the particle sizes and the surface modification affect the colour-switching speed. The high surface area of the small particles and low aggregation of the surface modification would increase the redox activity on the surface.

The *t*_B_ ratio of KZnHCF-MS to KZnHCF-BS, and 0.34. The particle size ratio of KZnHCF-MS to KZnHCF-BS were 0.34 and 0.42, respectively. The relation between the response speed and the particle size is reasonable because the specific surface area is inverse proportional to the particle size.

The cycle stabilities of the ECDs were evaluated for specifics with sequential step-like voltage change. The result shown in Fig. 4 (a) and (b) indicates that KZnHCF-MS and KZnHCF-M have longer cyclic stability than KZnHCF-BS, retaining 90% of the effective charge and 120% of the transmittance ratio to the initial values, respectively, in the oxidation reaction until one million cycles. After one million cycles, KZnHCF-MS shows similar transmittance in both redox states to that in the pre-cyclic test, as shown in Fig. 4(c).

Finally, the thermal influence on the ECD device was evaluated. An ECD of KZnHCF-MS was fabricated in same method of cycle test. The ECD in colour phase was kept in applying V = −1.3 V at 80 °C with evaluation of UV-vis. The absorbance at 680 nm was deceased from 0.85 to 0.52 for 21 hours as shown in ESI.† The result shows temperature has large influence on the memory effect of the ECD.

In summary, we developed the well-defined K_2_Zn_3_[Fe(CN)_6_]_2_·7H_2_O (KZnHCF)-NPs using a micromixer. The KZnHCF-NPs are transparent in both redox states and have high cyclic stability in the electrochemical redox reactions even after 1 million cycles. The non-change of the main framework of Zn-NC-Fe might be attributed to the cyclic stability of KZnHCF. Thus, it is a suitable candidate for application as a counter electrode in transparent-type ECDs.

## Conflicts of interest

There are no conflicts to declare.

## Supplementary Material

RA-009-C9RA09496B-s001
