# Supplementary material for: One million cyclable blue/colourless electrochromic device using K2Zn3[Fe(CN)6]2 nanoparticles synthesized with a micromixer
Source: RSC Adv. 2019 Dec 12;9(70):41083–7. doi: 10.1039/c9ra09496b (PMC9076391; doi:10.1039/c9ra09496b)
Supplement: RA-009-C9RA09496B-s001 [file RA-009-C9RA09496B-s001.pdf]

Electronic Supplementary Material (ESI) for RSC Advances.
This journal is © The Royal Society of Chemistry 2019

Electronic Supplementary Information (ESI)

One million cyclable blue/colourless electrochromic device using $K_2Zn_3[Fe(CN)_6]_2$ nanoparticles synthesized with a micromixer

Akira Takahashi, Hiroshi Watanabe, Keiko Noda, Tohru Kawamoto

Affiliation: Nanomaterials Research Institute, AIST, 1-1-1 Higashi, Tsukuba 305-8565, Japan

1. Synthesis optimisation

The influence of temperature in the micromixer synthesis is evaluated by the following experiment. The temperatures of the solutions before the micromixer was utilised were adjusted between 8 and 71 °C using a 10 m precooling tube consisting of an internal diameter (i.d.) of 0.5 mm SUS304. KZnHCF was synthesized by mixing 6 mmol/L of $ZnCl_2$ and 4 mmol/L of $K_4[Fe(CN)_6]$ aqueous solutions of which the pH was adjusted to 5. The total flow rates were set to 100 mL/min (v_L = 94 m/s) for 8 °C and 140 mL/min (v = 132 m/s) for the others.

The obtained KZnHCF's morphology was analysed using a scanning electron microscope (SEM, S-4800, Hitachi High Tech). The particle size was evaluated by SEM with the longest diagonal distance. The average particle size distribution was calculated with counting more than 100 particles per sample. The particle size decreased with the temperature, as shown in Fig. S1.

The actual particle size distribution of Fig. 1 (d) is shown in Fig. S2.

The SEM images of KZnHCF at different reagent concentrations are shown in Fig. S3.

The SEM image of bare-ITO on ITO-side is shown in Fig. S3.

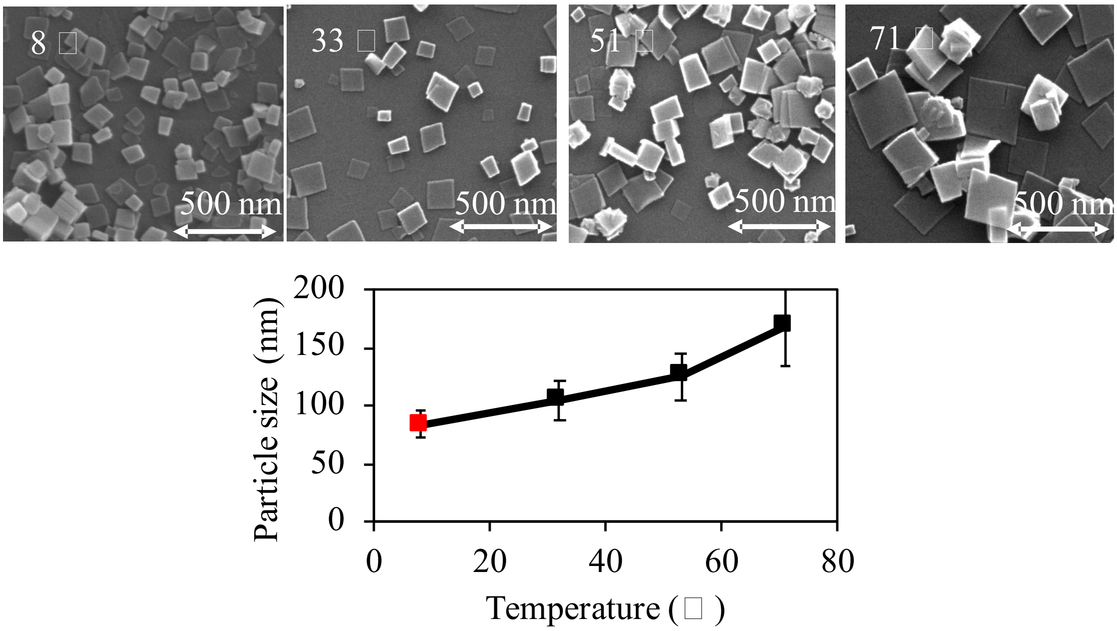

Figure S1. SEM images and the average size of KZnHCFs synthesized at different temperatures

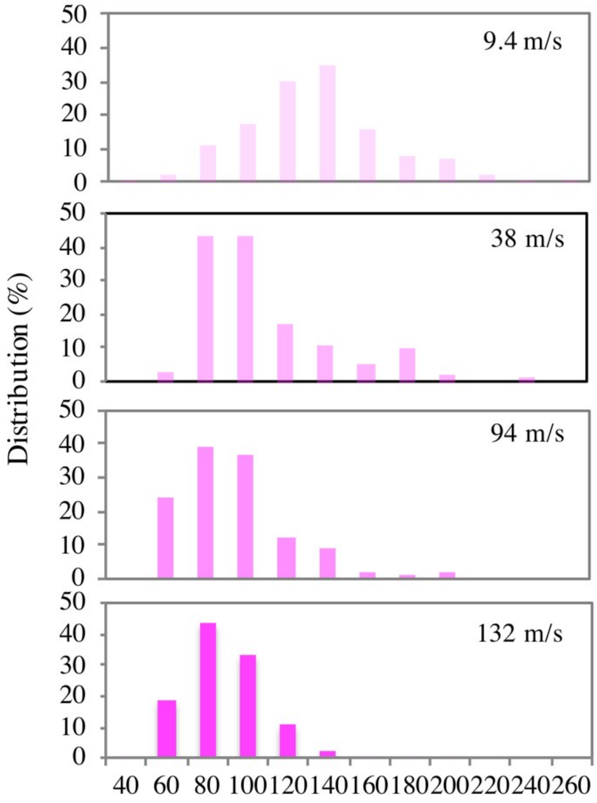

Figure S2. Particle size distribution of KZnHCF at different linear speeds

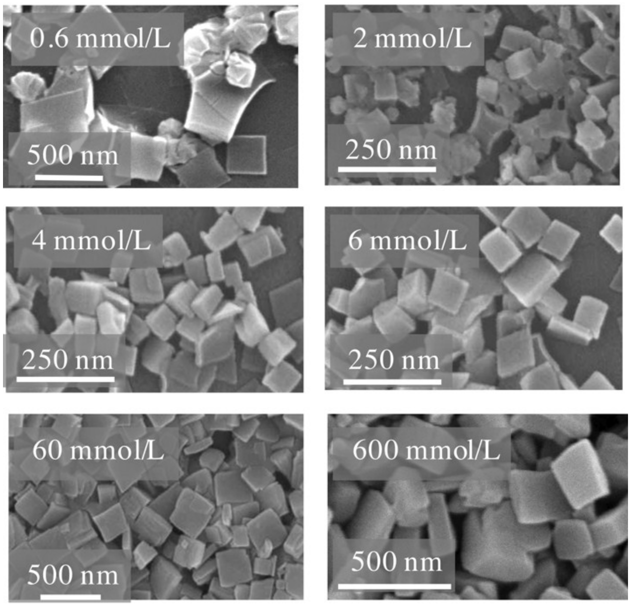

Figure S3. SEM images of KZnHCF synthesized with different concentrations

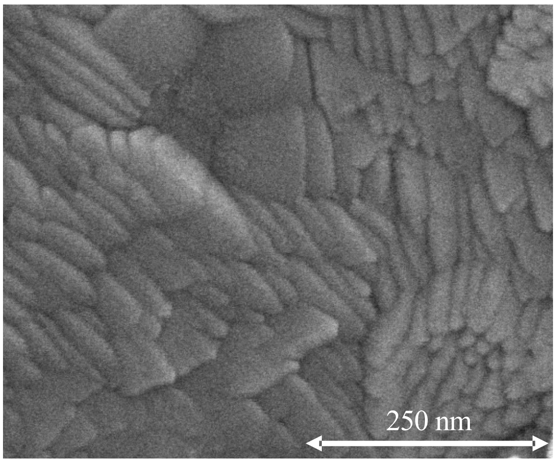

Figure S4. A SEM image of a bare ITO-glass on ITO-side.

2. Preparation of KZnHCF-M

The KZnHCF-suspended aqueous solution obtained by the standard condition of the KZnHCF micromixer synthesis written in the main manuscript was purified with the following process to remove by-products (mainly KCl). The KZnHCF-suspended aqueous solution, set in 6 centrifuge tubes with a volume of 45 mL, was centrifuged under the condition of 16,000 G for 30 min. The supernatant water was removed from the tube, and the same amount of the KZnHCF-suspended aqueous solution was added into the tubes. After the concentration of KZnHCF from 1 L of the solution into 6 tubes, KZnHCF was purified by centrifugation under the condition of 16,000 G for 30 min, and the supernatant and ultrapure water were replaced 5 times. After purification, the KZnHCF powder was obtained after drying the suspended solution using an evaporator under the temperature and pressure conditions of

60 °C and 60 hPa. The sample is referred to as KZnHCF-M (M: synthesized with micromixer).

3. Preparation of KZnHCF-MS

The purified KZnHCF suspended solution was prepared using the process for the KZnHCF-M sample. Afterwards, the purified KZnHCF sample was mixed with 7 mL of a 6 mmol/L $K_4[Fe(CN)_6]$ aqueous solution, and the suspended aqueous solution was dispersed with an ultrasonic cleaner for 1 h. After the dispersion process, the KZnHCF powder was obtained after the suspended solution was dried with an evaporator under the conditions of 60 °C and 60 hPa. The sample is referred to as KZnHCF-MS (M: synthesized with micromixer, S: surface modification).

4. Preparation of KZnHCF-B

The KZnHCF suspended aqueous solution was prepared by mixing 1.5 L of 6 mmol/L $ZnCl_2$ and 1.5 L of 4 mmol/L of $K_4[Fe(CN)_6]$ and cooling the solution between 5 and 10 °C. Using the KZnHCF-suspended aqueous solution, the KZnHCF powder was obtained using the same purification and drying processes of KZnHCF-M. The sample is referred to as KZnHCF-B (B: synthesized through the batch process).

5. Preparation of KZnHCF-BS

The purified KZnHCF suspended solution was prepared using the process of the KZnHCF-B sample. Afterwards, the purified KZnHCF sample was mixed with 7 mL of 6 mmol/L $K_4[Fe(CN)_6]$ aqueous solution, and the suspended aqueous solution was dispersed with an ultrasonic cleaner for 1 h. After the dispersion process, the KZnHCF powder was obtained after the suspended solution was dried using an evaporator under the conditions of 60 °C and 60 hPa. The sample is referred to as KZnHCF-BS (B: synthesized through the batch process, S: surface modification).

6. Chemical composition of the KZnHCFs

The metal cation proportion in the chemical composition of KZnHCFs was determined using an inductively coupled plasma mass spectrometer (ICP-MS, NexION 300D Perkin Elmer Co., Ltd.) with a prior decomposition system (Multiwave 3000 Perkin ElmerCo., Ltd.). The water quantity of KZnHCFs was determined by thermogravimetry differential thermal analysis (TG-DTA, Thermo plus evo II, Rigaku Corp.). The results are shown in Table S1.

Table S1. Chemical compositions of KZnHCFs

Sample	Composition Formula	K (wt%)	Zn (wt%)	Fe (wt%)	H_2O (wt%)
**KZnHCF (calculated)**	$K_{2.0}Zn_{3.0}[Fe(CN)_6]_{2.0}\cdot 7.0H_2O$	9.5	23.8	13.8	15.3
**KZnHCF-M**	$K_{1.8}Zn_{3.0}[Fe(CN)_6]_{1.9}\cdot 6.9H_2O$	8.6	23.9	13.1	15.2
**KZnHCF-MS**	$K_{1.9}Zn_{3.0}[Fe(CN)_6]_{1.9}$ $7.2H_2O$	9.0	23.8	13.1	15.7
**KZnHCF-BS**	$K_{2.0}Zn_{3.0}[Fe(CN)_6]_{2.0}\cdot 6.8H_2O$	9.7	24.3	13.5	15.2

7. PXRD for crystal structure evaluation

The KZnHCF-MS suspended aqueous solution was spin-coated under the conditions of 800 rpm for 10 s and 2000 rpm for 10 s on a stainless substrate (SUS304) hydrophilised using a plasma-cleaner (PDC-001-HP, Harrick Plasma Inc.) for 3 min. The initial reduction state of KZnHCF was evaluated as the film without electrochemical treatment. In the case of measuring the oxidation state, the films were put in an electrolyte of propylene carbonate (PC) solution containing 0.1 mol/L(PC) of potassium bis(trifluoromethanesulfonyl) imide with 3-terminal method consisting of a saturated calomel electrode (SCE) as the reference electrode, and platinum wire as the counter electrode. The electrochromic behaviour was evaluated using an electrochemical analyzer (ALS6115D BAS Inc.). After oxidising the KZnHCF-MS thin film by adding 1.4 V vs SCE for 2 min, it was removed from the electrolyte solution and measured by PXRD (Fig. ESI 4(a)).

The crystal structure of KZnHCF consists of a porous Zn-NC-Fe network with tetrahedral nitrogen coordinated to Zn^{2+} cation and octahedral carbon coordinated to Fe^{2+} (Fig. ESI 5(b)). Potassium cation and water molecules exist in the porous network. The pore size of each axes are shown in Fig S6(a),(b) and (c). All pore sizes are larger than ionic diameter of monovalent potassium cation, 1.37 ~ 1.64 Å (R. D. Shannon, Acta. Cryst., 1976 **A32**, 751). The pore cross-section size of both a-axis and b-axis were 21, 2 Å^2 and that of c-axis was 28.1 Å^2 with the approximation of Rhomboid.

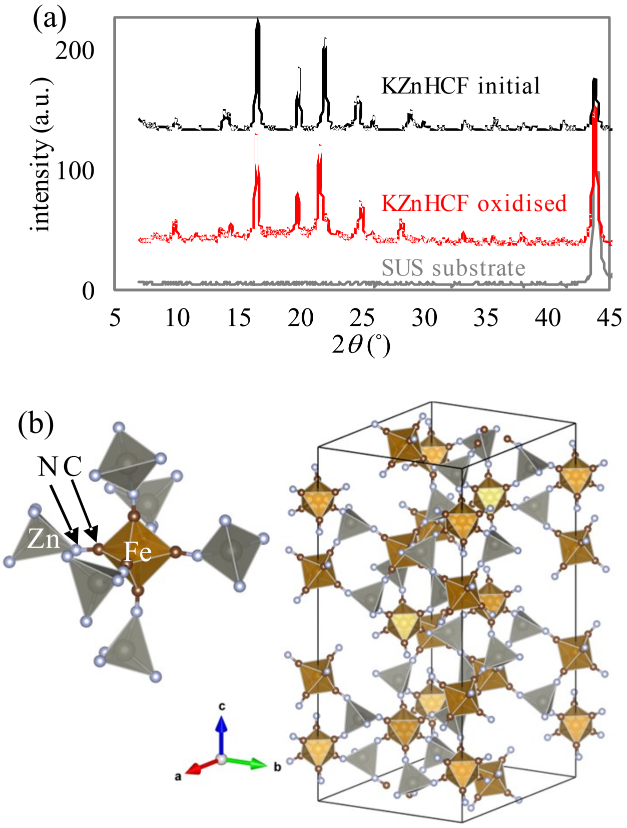

Figure S5 (a) PXRD pattern of the initial and oxidised state of KZnHCF, (b) crystal structure of $K_2Zn_3[Fe(CN)_6]_2$.

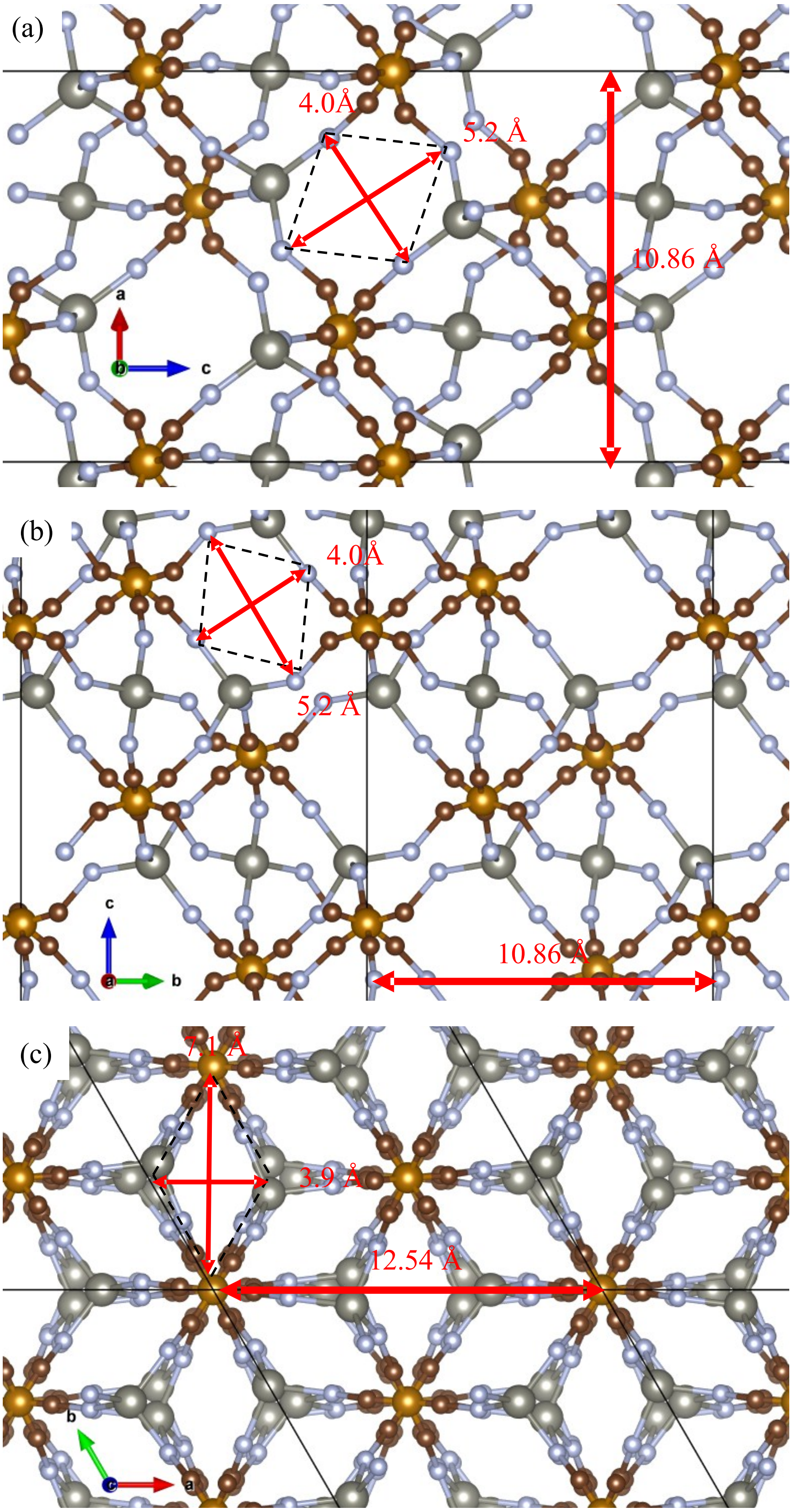

Figure S6 the pore size of KZnHCF in (a) a-axis, (b) b-axis and (c) c-axis.

8. Fabrication of ECDs

Blue/colourless switching ECDs were fabricated using a Prussian blue film and KZnHCFs. The ECD contained an electrolyte sandwiched between the Prussian blue film on an ITO glass and the KZnHCF-MS film on the ITO glass with these films facing the internal electrolyte.

The Prussian blue films were prepared as follows: a Prussian blue ($Fe^{III}[Fe^{II}(CN)_6]_{3/4}$) suspended aqueous solution was purchased from Kanto Chemical co., inc. The Prussian blue 6.3 wt% aqueous solution was spin-coated on the ITO glass hydrophilised with a plasma-cleaner for 3 min. The condition of the spin-coating was *R* rpm for 10 s and *R*+500 rpm for 10 s with a changing rotation speed *R* (rpm) for adjusting the UV-vis transmittance at 700 nm between 15 and 23%. The R of KZnHCF-B, KZnHCF-M, KZnHCF-MS were 1500, 1200-1300, and 1000 respectively.

An electrochemical pre-treatment of the Prussian blue films was performed before the fabrication of the ECD. The films were put into an electrolyte of propylene carbonate (PC) solution containing 0.1 mol/L(PC) of potassium bis(trifluoromethanesulfonyl) imide with 3-terminal method consisting of saturated calomel electrode (SCE) as the reference electrode, and platinum wire as the counter electrode. The electrochromic behaviour was evaluated using an electrochemical analyzer (ALS6115D BAS inc.). The sequential step-like change of the potential was performed with the following potential and time of -0.4 V (v.s. SCE) for 90 s, 0.6 V (v.s. SCE) for 90 s, and 1.2 V (v.s. SCE) for 240 s.

The KZnHCF films were prepared as follows: aqueous solutions of KZnHCF-M, KZnHCF-MS, and KZnHCF-BS were spin-coated on the ITO glass. The concentrations of KZnHCF-M and KZnHCF-MS were 10 wt% and that of KZnHCF-BS was 20 wt%. The KZnHCF films were fabricated by the spin-coating method similar to that for the Prussian blue films for adjusting the electrochemical charge of the films between 12 and 18 mC/cm^2.

An electrochemical pre-treatment of the KZnHCF films was performed before the fabrication of the ECD in the same setting as that of the Prussian blue film. A CV curve was obtained under the following conditions: a sweeping speed of 5 mV/s, a start potential of 0.4 V, a middle potential of 1.4 V, and a final potential of 0.4 V (v.s. SCE). After the CV, the film was maintained at 0.4 V (v.s. SCE) for 300 s.

The ECDs were fabricated by sandwiching the PC electrolyte containing 0.1 mol/L(PC) of potassium bis(trifluoromethanesulfonyl) imide and 360 g/L(PC) of PMMA. The side of the electrolyte was sealed with a UV-resin (X-DS3V, kyouritsu kagaku). Five ECDs were fabricated with Prussian blue films and KZnHCF films.

9. reaction speed of ECDs

The reaction speeds of the ECDs were evaluated with monitoring transmittance ratio to initial value at 700 nm, Resulting the Fig. S7.

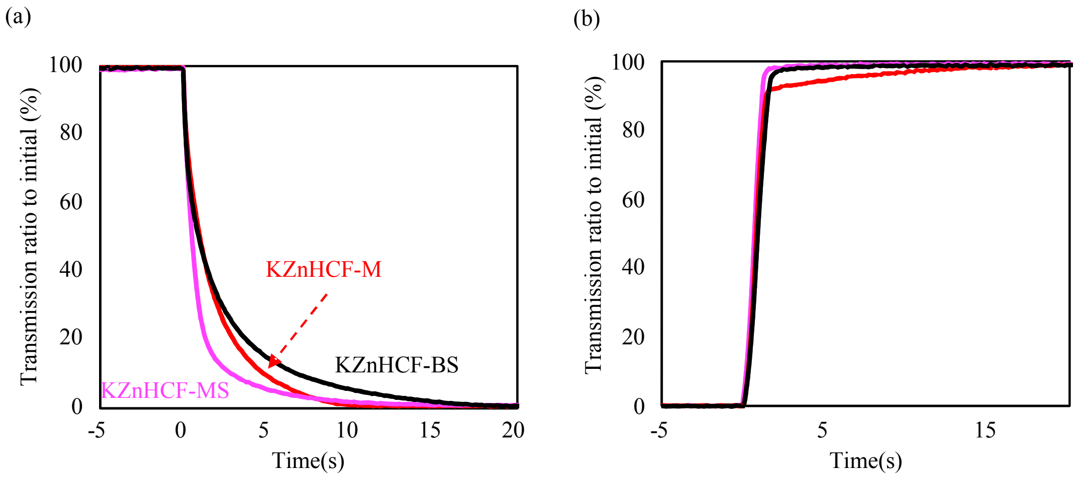

Figure S7 the reaction speeds of ECDs at (a) bleaching and (b) colouring process.

10. Thermal stability of ECDs

The thermal stability of the ECDs fabricated with above method using KZnHCF-MS was evaluated. The ECD was put in electric furnace at 80˚C with adding -1.3V continuously. The UV-vis spectra of sample observed with adding -1.2V at initial state and after 1, 5, and 21 hours as shown in Fig S8.

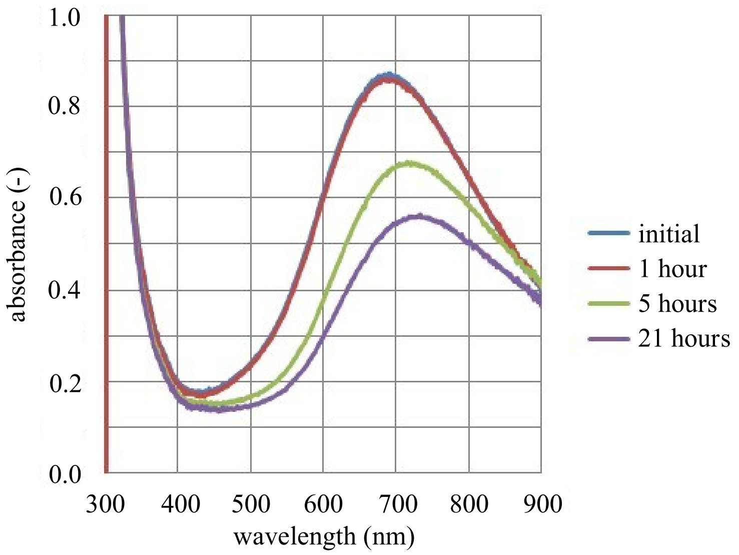

Figure S8 UV-vis spectra of ECD after each time.
